# An Antibiogram Study for Urine Culture Testing in Makkah Region Hospitals

**DOI:** 10.7759/cureus.36012

**Published:** 2023-03-11

**Authors:** Anas S Dablool

**Affiliations:** 1 Public Health Department, Faculty of Health Sciences, Umm Al-Qura University, Makkah, SAU

**Keywords:** urinary tract, resistant bacteria, makkah, urine culture, antibiogram

## Abstract

Background: The antibiogram profile could be helpful in the selection of the most appropriate antimicrobial treatment for microbial infection and even useful to monitor antibiotic resistance. Objective: This study aims to identify the bacteria in the urine through urine culture and perform their antibiogram to determine the resistance profile between antibiotics and urine tract infection (UTI)-causing bacteria and to determine the effective and non-effective antibiotics. Methods: The study was based on urine culture data from five Makkah hospitals in the Kingdom of Saudi Arabia (KSA). Results: A total of 1000 pathogens were found in the urine culture; 899 were bacterial isolates, and 101 were *Candida spp*. Seven hundred and seventy-seven of the 899 bacteria isolates were gram-positive, while 122 were gram-negative bacteria. *Escherichia coli* (44%) was the most frequent UTI-causing bacteria, followed by *Klebsiella spp.* (20%), *Pseudomonas aeruginosa* (6%), *S. aureus* (5.5%), *Enterococcus faecalis* (4.5%), *Enterobacter spp.* (2%), and *Proteus spp.* (1%). There was clear evidence that ampicillin, cefepime, erythromycin, and moxifloxacin were not effective antibiotics for uropathogens in the Makkah area, KSA. The multiple drug resistance (MDR), extensively drug-resistant (XDR), extended spectrum beta lactamase (ESBL), CR, and quinolones resistance (QR) were higher in the gram-negative bacilli. The pandrug resistance (PDR) and AmpC seemed to have fewer ratios of UTIs caused by gram-negative bacteria. On the other hand, *S. aureus* of the gram-positive type was also involved in the UTI and had a higher ratio of MDR, QR, and methicillin-resistant Staphylococcus aureus (MRSA).

## Introduction

UTIs are frequent illnesses that develop when bacteria enter the urethra and infect the urinary system. These germs are frequently found on the skin or rectum. Although the infections can affect different regions of the urinary system, a bladder infection (cystitis) is the most prevalent. Another kind of UTI is a kidney infection, often known as pyelonephritis [1]. Urine testing is the main element in diagnosing an UTI. It includes collecting midstream urine with an aseptic method followed by urine culture, which involves identification of the causative pathogen and sensitivity testing. Urine dipsticks and urine microscopy are two of the most frequently used methods of diagnostic testing, especially when there is suspicion that a patient is suffering from an UTI [1,2]. In the urinary tract, bacteria enter through the urethra and grow rapidly to develop an infection. Generally, the infection begins in the urethra or bladder, but it has the ability to spread to other parts of the system [2-4].

Multiple drug resistance (MDR) is bacterial antimicrobial resistance to at least one antimicrobial drug in three or more antimicrobial categories. Extensively drug-resistant (XDR) is defined as nonsusceptibility to at least one agent in all but two or fewer antimicrobial categories. Pandrug Resistance (PDR) is the non-susceptibility to all agents in all antimicrobial categories (i.e., bacterial isolates are not susceptible to any clinically available drug). An antibiogram is an overall profile of antimicrobial susceptibility testing results for a specific microorganism to many antimicrobial drugs. This profile is generated by the laboratory using aggregate data from a hospital or healthcare system; the data are summarized periodically and presented, showing percentages of organisms tested that are susceptible to a particular antimicrobial drug. Only results for antimicrobial drugs that are routinely tested and clinically useful should be presented to clinicians. The overall antimicrobial susceptibility testing profile of any microorganism to a series of anti-microbial drugs is termed an antibiogram [5]. The laboratory obtains the data from a healthcare system or hospital and produces that profile by aggregating the data. It shows the ratio of the organism that is susceptible to a specific type of drug. This antibiogram profile helps the pharmacist and clinician to select the most appropriate antimicrobial treatment for a microbial infection. This tool is quite useful to detect and monitor antibiotic resistance [6-9]. This is a research-based study in which the urine culture antibiogram has been generated with urine pathogens against the different antibiotics in Makkah city hospitals, KSA, and the antibiotic resistance, as well as the most MDRs of different types of urine pathogens, have been discussed.

## Materials and methods

Data collection

This retrospective study was performed using data from the Saudi hospitals in the Makkah region through a surveillance network for antimicrobial resistance (AMR) in pathogenic bacteria in the urine. In this study, urine culture data has been collected in outpatient clinics and inpatient departments from five tertiary hospitals in Makkah, KSA, during the period of 1 January 2020 to 31 December 2020. Data collection, including demographic data (age, gender, education, and type of pathogen), was performed manually from the databases of the two departments of the laboratory and medical records corresponding to urine samples that were positive for urine pathogens. A total of 1000 non-duplicated urine pathogens from 1000 patients were identified in hospitals during the study period. Most of the patients were males: 641 (64.1%), while the females were 359 (35.9%). The majority of patients with UTI were above 60 years old, representing 351 (35.1%) of the total number of patients, whereas 249 (24.9%) of the patients were between 41 and 50 years, 200 (20%) were between 51 and 60 years, 148 (14.8%) were between 20 and 40 years, whereas 62 (6.2%) patients were younger than 20 years.

Bacterial identification, antimicrobial susceptibility testing

The processing of urine samples arriving at the laboratory departments (Medical Microbiology) during the study period was done by identification of isolates using classical biochemical tests, standard bacteriological cultures, and methods for bacterial isolation and identification. Antibiograms are performed using both the disk diffusion method and direct AST by the VITEK-2 (bioMérieux, Marcy l'Étoile, France) compact system. All protocols were performed according to the manufacturer’s instructions. VITEK cards AST-GN13, AST-GN09, AST-GP67, containing Penicillin-G, ampicillin, tigecycline, Ciprofloxacin, Levofloxacin, Moxifloxacin, Erythromycin, Clindamycin, Vancomycin, linezolid, Sulfamethoxazole, Amoxicillin-Clavulanic acid, Tobramycin, Amikacin, Gentamicin, Nitrofurantoin, Oxacillin, Piperacillin-Tazobactam, Cephalothin, Ceftriaxone, Ceftazidime, Cefepime, Meropenem, Imipenem, and Colistin were used for Enterobacteriaceae, gram-negative bacilli, and gram-positive cocci. Isolates were then categorized as susceptible, intermediate, or resistant.

Data analysis

The data has been used in the laboratory to develop the antibiogram for all of the mentioned bacteria against different antibiotic variables extracted, including bacterial species, susceptibility categories (susceptible or resistant) to each tested antibiotic, and resistant threats such as MDR, XDR, PDR, ESBL, AmpC, Carbapenemase Resistance (CR), MRSA, Vancomycin-Resistant Enterococcus (VRE), and Quinolones Resistance (QR). After getting the antibiogram from the laboratory test, a chart has been developed showing the profile of all the antibiotics against the bacteria in percentage.

## Results

The result of the present study showed that there were 13 gram-negative bacilli (*E. coli*, *Klebsiella spp.*, *Proteus spp.*, *Enterobacter spp.*, *Citobacter spp.*, *Serratia marcescens*, *Morganella*, *Providencia spp.*, *Salmonella spp.*, *P. aeruginosa*, *Acinetobacter*, *Burkholderia cepacian*, *N. gonorrhoeae*) and six gram-positive cocci (namely, *S. aureus*, *S. saprophyticus*, *S. haemolyticus*, *Enterococcus faecalis*, *E. faecium*, and *S. agalactiae*) one candida isolate have been isolated during the studied period. During the Urine Cultural Test, there were 1000 total pathogens found, out of which 101 were *Candida spp.*, while the remaining 899 were bacterial isolates, which are further divided into gram-negative and gram-positive, 777 and 122, respectively. Table 1 presents the urine cultural bacterial isolates.

**Table 1:** Urine Culture Data of Bacterial Species Isolated From Urine Culture in Hospitals of the Makkah Region

Organism	No.	%
E. coli	442	44
Klebsiella spp.	197	20
Proteus spp.	11	1
Enterobacter spp.	24	2
Citrobacter spp.	9	1
Serratia marcescens	5	0.5
Morganella	4	0.4
Providencia spp.	4	0.4
Salmonella spp.	2	0.2
T. Enterobacteriaceae	698	70
P. aeruginosa	57	5.7
Acinetobacter	19	2
Burkholderia cepacia	2	0.2
N. gonorrhoeae	1	0.1
Total gram-negative bacilli	777	78
S. aureus	53	5.3
S. saprophyticus	8	0.8
S. haemolyticus	4	0.4
Enterococcus faecalis	44	4.4
E. faecium	5	0.5
Total enterococci	49	4.9
S. agalactiae	9	1
Total gram-positive cocci	122	12
Total bacterial isolates	899	90
Candida spp.	101	10
Total pathogens	1000	100

The most prevalent species within the gram-negative bacilli were *E. coli*, with a ratio of 44% (442), followed by the *Klebsiella spp.*, having a ratio of 20% (197), *Enterobacter spp.*, 2% (24), *Citrobacter spp.*, 1% (9), and *Proteus spp.* 1% (11). In addition to these, *Serratia marcescens* is also found with a portion of 0.5% (5), *Morganella* and *Providencia spp.* both have a ratio of 0.4% (4), and the *Salmonella spp.* have a ratio of 0.2% (2). In this way, there are a total of 70% *Enterobacteria* in the 78% of gram-negative bacteria. The most common species were *P. aeruginosa*, with 5.7% (57), *Acinetobacter*, with a ratio of 2% (19), *Burkholderia cepacia* with a ratio of 0.2% (2), and *N. gonnorrhoae*, with a 0.1% (1) ratio.

The second major part of the bacterial isolate was gram-positive cocci, which is mainly based on the *S. aureus*, which has a ratio of 5.3% (53), followed by *Enterococcus faecalis* with a ratio of 4.4% (44), followed by the *E. faecium*, which has a ratio of 0.5% (5), *S. saprophiticus* with 0.8% (8) of the total gram-positive, the *S. heamolyticus* is 0.4% (4), and the *S. agalactae* is 1% (9) of the total gram-positive *cocci*.

Susceptibility results

The result of antimicrobial susceptibility testing showed that 100% of gram-positive bacteria were sensitive to vancomycin and linezolid, followed by levofloxacin (90%) and tigecycline (82%), as shown in Table 2. It was found that 42.6% of gram-positive bacteria were resistant to oxacillin, while 34.4% of them were resistant to moxifloxacin and erythromycin (Table 2).

**Table 2:** Antimicrobial Susceptibility Testing Results of Gram-Positive Cocci

Total number (122)	Sensitive		Intermediate		Resistant	
	No	%	No	%	No	%
Penicillin-G	99	81.1	3	2.5	20	16.4
ampicillin	95	77.9	15	12.3	12	9.8
tigecycline	100	82	5	4.1	17	13.9
Levofloxacin	110	90.2	7	5.7	5	4.1
Moxifloxacin	68	55.8	12	9.8	42	34.4
Erythromycin	65	53.3	15	12.3	42	34.4
Clindamycin	77	63.1	13	10.7	32	26.2
Vancomycin	122	100	0	0	0	0
Linezolid	122	100	0	0	0	0
Sulfamethoxazole	91	74.6	9	7.4	22	18
Amoxc+Clavunate	63	51.6	42	34.4	17	14
Gentamicin	95	77.9	3	2.5	24	19.6
Nitrofurantoin	98	80.3	8	6.6	16	13.1
Oxacillin	60	49.2	10	8.2	52	42.6

The results also showed that 99.7% of gram-negative bacteria were sensitive to colistin, followed by meropenem (96.5%) and imipenem (94%), as shown in Table 3. It was found that 56.5% of gram-negative bacteria were resistant to ampicillin, while 45.6% of them were resistant to cefepime (Table 3).

**Table 3:** Antimicrobial Susceptibility Testing Results of Gram-Negative Bacilli

Total number (777)	Sensitive		Intermediate		Resistant	
	No	%	No	%	No	%
Ampicillin	246	31.7	92	11.8	439	56.5
Amoxicillin-Clavulanic acid	356	45.8	230	29.6	191	24.6
Piperacillin-Tazobactam	511	65.8	120	15.4	146	18.8
Cephalothin	416	53.5	300	38.6	61	7.9
Ceftriaxone	520	67	120	15.4	137	17.6
Ceftazidime	550	70.8	210	27	17	2.2
Cefepime	335	43.1	88	11.3	354	45.6
Gentamicin	632	81.3	101	13	44	5.7
Amikacin	740	95.2	10	1.3	27	3.5
Ciprofloxacin	420	54	225	29	132	17
Tigecycline	695	89.4	73	9.4	9	1.2
Nitrofurantoin	220	28.3	300	38.6	257	33.1
Sulfamethoxazole	445	57.3	115	14.8	217	27.9
Meropenem	750	96.5	5	0.7	22	2.8
Imipenem	730	94	10	1.3	37	4.7
Levofloxacin	655	84.3	22	2.8	100	12.9
Tobramycin	610	78.5	141	18.2	26	3.3
Colistin	775	99.7	2	0.3	0	0

Resistant threats within total gram-negative bacilli

Table 4 shows that 47% of *Enterobacteriaceae* were MDR. *E. coli* showed the highest MDR (51%), followed by *Klebsiella spp.* (44%), *Proteus spp.* (27%), *Enterobacter spp.* (22.6%), *Acinetobacter* (0.4%), and *P. aeruginosa* (0%).

It found that 4.9% of *Enterobacteriaceae* were XDR. *Klebsiella spp.* showed the highest XDR (27%), followed by *Acinetobacter* (20%) and *Enterobacter spp.* (3%), *P. Aeruginosa* (2.7%), and *E. coli* (0.5%). The PDR in *Klebsiella spp.* showed the highest ratio (3.5%), while the *E. coli* ratio was 0.2%, while *Proteus spp.*, *Enterobacter spp.*, *P. aeruginosa*, and *Acinetobacter* had 0% PDR.

The highest ratio of ESBL was shown by *Proteus spp.* (53%), followed by *Klebsiella spp.* (40%) and *E. coli* (39%), and the least was made by *Enterobacter spp.* (22.6%).

Also, the results showed that about 13% of the *Enterobacter spp.* and 0.7% of *E. coli* were AmpC.

Most of the species of *Enterobacteriaceae* were CR except *E. coli* (0.3%). On the other hand, *Klebsiella spp.*, *Proteus spp.*, and *Enterobacter spp.* showed a significant ratio of CR, which was 10.5%, 13%, and 3%, respectively.

Resistant threats within total gram-positive bacilli

Only *S. aureus* and *Enterococcus faecalis* showed a ratio of 39% and 5.3% as an MDR; no other species showed an MDR. Only two species showed QR, mainly *S. aureus* (27.5%) and *Enterococcus faecalis* (24.6%). Other anti-biotic resistance during the antibiogram of urine culture. The XDR was shown in total *enterococci* with a ratio of 9.5%. Similarly, VRE was shown with a ratio of 3.2%. and MRSA was 39% in *S. aureus*.

**Table 4:** Resistant Threats Within Bacterial Isolates From Urine Cultures

Organism	No.	%	MDR		XDR		PDR		ESBL		AMPC		CR		QR		MRS		VRE	
			No.	%	No.	%	No.	%	No.	%	No.	%	No.	%	No.	%	No. %		No. %	
E. coli	442	44.2%	227	51.4%	2	0.5%	1	0.2%	171	38.7%	3	0.7%	2	0.5%	184	41.6%				
Klebsiella spp.	197	19.7%	86	43.7%	23	11.7%	7	3.6%	78	39.6%	0	0.0%	21	10.7%	77	39.1%				
Proteus spp.	11	1.1%	3	27.3%	3	27.3%	0	0.0%	6	54.5%	0	0.0%	2	18.2%	5	45.5%				
Enterobacter spp.	24	2.4%	5	20.8%	1	4.2%	0	0.0%	5	20.8%	3	12.5%	1	4.2%	4	16.7%				
Citrobacter spp.	9	0.9%	2	22.2%	1	11.1%	0	0.0%	2	22.2%	0	0.0%	1	11.1%	2	22.2%				
Serratia marcescens	5	0.5%	3	60.0%	0	0.0%	0	0.0%	3	60.0%	0	0.0%	2	40.0%	2	40.0%				
Morganella	4	0.4%	2	50.0%	0	0.0%	0	0.0%	2	50.0%	0	0.0%	0	0.0%	2	50.0%				
Providencia spp.	4	0.4%	0	0.0%	1	25.0%	0	0.0%	1	25.0%	0	0.0%	1	25.0%	1	25.0%				
Salmonella spp.	2	0.2%	2	100.0%	0	0.0%	0	0.0%	1	50.0%	1	50.0%	0	0.0%	2	100.0%				
T. enterobacteriaceae	698	69.8%	330	47.3%	31	4.4%	8	1.1%	269	38.5%	7	1.0%	30	4.3%	279	40.0%	0	0.0%	0	0.0%
P. aeruginosa	57	5.7%	0	0.0%	2	3.5%	0	0.0%	0	0.0%	0	0.0%	8	14.0%	2	3.5%				
Acinetobacter	19	1.9%	1	5.3%	4	21.1%	0	0.0%	0	0.0%	0	0.0%	4	21.1%	4	21.1%				
Burkholderia cepacia	2	0.2%	0	0.0%	2	100.0%	0	0.0%	0	0.0%	0	0.0%	0	0.0%	2	100.0%				
N. gonorrhoeae	1	0.1%	0	0.0%	0	0.0%	0	0.0%	0	0.0%	0	0.0%	0	0.0%	0	0.0%				
Total gram-negative bacilli	777	77.7%	331	42.6%	39	5.0%	8	1.0%	269	34.6%	7	0.9%	42	5.4%	287	36.9%	0	0.0%	0	0.0%
S. aureus	53	5.3%	21	39.6%	0	0.0%	0	0.0%	0	0.0%	0	0.0%	0	0.0%	15	28.3%	21	39.6%	0	0.0%
S. saprophyticus	8	0.8%	2	25.0%	0	0.0%	0	0.0%	0	0.0%	0	0.0%	0	0.0%	0	0.0%	2	25.0%	0	0.0%
S. haemolyticus	4	0.4%	2	50.0%	0	0.0%	0	0.0%	0	0.0%	0	0.0%	0	0.0%	2	50.0%	2	50.0%	0	0.0%
Enterococcus faecalis	44	4.4%	2	4.5%	0	0.0%	0	0.0%	0	0.0%	0	0.0%	0	0.0%	11	25.0%	0	0.0%	0	0.0%
E. faecium	5	0.5%	0	0.0%	5	100.0%	0	0.0%	0	0.0%	0	0.0%	0	0.0%	5	100.0%	0	0.0%	2	40.0%
Total enterococci	49	4.9%	2	4.1%	5	10.2%	0	0.0%	0	0.0%	0	0.0%	0	0.0%	16	32.7%	0	0.0%	2	4.1%
S. agalactiae	9	0.9%	0	0.0%	0	0.0%	0	0.0%	0	0.0%	0	0.0%	0	0.0%	2	22.2%	0	0.0%	0	0.0%
Total gram-positive cocci	122	12.2%	27	22.1%	5	4.1%	0	0.0%	0	0.0%	0	0.0%	0	0.0%	35	28.7%	25	20.5%	2	1.6%
Total bacterial isolates	899	89.9%	358	39.8%	44	4.9%	8	0.9%	269	29.9%	7	0.8%	44	4.9%	322	35.8%	25	2.8%	2	0.2%
Candida spp.	101	10.1%																		
Total pathogens	1000	100%																		

## Discussion

The present study has generated a urine culture antibiogram, including pathogens and their sensitivities against the different antibiotics in Makkah city hospitals. The result showed that the antibiogram of the urine culture was an indication of the antibiotic resistance of several microbial species and their effectiveness. The study showed that 100% of gram-positive bacteria were sensitive to vancomycin and linezolid, while 42.6% and 34.4% of them were resistant to oxacillin and erythromycin/moxifloxacin, respectively. In addition, the study also showed that 99.7% of gram-negative bacteria were sensitive to colistin, followed by meropenem (96.5%) and imipenem (94%), while 56.5% and 45.6% of them were resistant to ampicillin and cefepime, respectively (Table 3).

The prevalence of MDR was 51%, followed by ESBL (39%) and QR (42%), which were not significant because bacteria have great resistance to them, while XDR, PDR, AmpC, and CR could also emerge in E. coli. Another gram-negative bacillus (*Klebsiella spp.*) showed a similar profile and was higher at MDR (44%), followed by ESBL (40%), QR (39%), XDR (12%), and CR (10.5%). Only the PDR and AMPC can be expressed in *Klebsiella spp.* *Proteus spp.* has also been found to show expression of ESBL (53%), QR (47%), MDR & XDR (27%), and CR (10.5%). The PDR and AMPC had been reported prevalent in *Proteus spp.* [10-12].

Furthermore, the present study found that the Enterobacter spp. were MDR (22.6%), ESBL (22.6%), AMPC (13%), and QR (16%), except that all the antibiotics can be effective [13]. In addition, *P. aeruginosa* and *Acinetobacter* were both found as QR, while *Acinetobacter* was found to be XDR and QR simultaneously. In this connection, all the antibiotic resistance except the CR can be found in *P. aeruginosa*. However, other gram-negative bacilli, including *Citrobacter spp.*, *Serratia marcescens*, *Morganella*, *Providencia spp.*, *Salmonella spp.*, *Burkholderia cepacia*, and *N. gonorrhoeae*, showed no resistance to any of the antibiotics, so it can be concluded that all of the mentioned antibiotic resistance may be found in these microbes [14].

For the gram-positive *cocci*, the present study found that *S. aureus* was the most MDR (39%), MRSA (39%), and QR (27.5%). All the other antibiotics are good for this microbe. After that, *Enterococcus faecalis* was found at QR (24.6%). Besides these two, all the *cocci* can be treated with any of the previously mentioned antibiotics.

In our study, we found *E. coli* to be the most frequent microbial that causes urinary tract infection (UTI). A similar result has been found by Gessese et al. [15]. They conducted a study on “Urinary pathogenic bacterial profile, antibiogram of isolates, and associated risk factors among pregnant women in Ambo town, Central Ethiopia: a cross-sectional study,” where they studied 300 urine samples and found *E. coli* to be the most frequent microbial with a ratio of 46.6%. There are also some similarities in the microbes between our study and the study by Gessese et al. [10]. They found *Proteus spp.* and *S. aureus* too, in their study, just like ours. Gessese et al. also found a similarity with the most resistant antibiotic among gram-negative bacilli; in their study, the MDR found most of the resistant antibiotics among gram-negative bacteria, and our study also found similar findings.

A study is also made by Kibret and Abera [4]. They have made the antibiogram study on 1404 urine samples from Ethiopia. They have found that the most frequent and most infection-causing bacilli are named *E. coli*, with a ratio of 63.6%, followed by *Klebsiella spp.* and *Proteus spp.* This study also has similar findings to ours, not for the prevalence of microbes but for the resistance to antibiotics. They have noticed that the gram-negative bacteria are showing great resistance to MDR.

There are studies [16-20] with similar results to our study. Most studies found the most frequent bacilli to cause UTIs to be *E. coli*, *Klebsiella spp.*, and *Proteus spp.* Most of them are related to the gram-negative type of bacteria, and they show high MDR.

Limitations and implications

This study is based on urine samples obtained from hospitals in the Makkah region, which sets a major limitation on the implication of this study. The antibiogram is showing the profile of the microbial and their possible treatment with antibiotics for a specific region of Makkah city. The result of this study did not represent the KSA country or other countries of the world. Similar studies are recommended to be undertaken in the other regions and cities of KSA.

## Conclusions

The study showed clear evidence that ampicillin, cefepime, erythromycin, and moxifloxacin are not effective antibiotics for uropathogens in the Makkah area, KSA. The MDR, XDR, ESBL, CR, and QR were higher in the gram-negative bacteria. The PDR and AmpC seemed to have fewer ratios of UTI caused by gram-negative bacteria. On the other hand, S. aureus of the gram-positive type was also involved in the UTI and had a higher ratio of MDR, QR, and MRSA.
